# Progress in Surgery

**Published:** 1898-11-05

**Authors:** 


### PROGRESS IN SURGERY.

### SURGERY OF THE LIVER, GALL-BLADDER, AND SPLEEN.

**Hepatic Tumours** are considered by Terrier and Auvray,[1] who think that the opportunities afforded to the surgeon of intervening in such instances must be regarded as very rare, because in most cases of hepatic tumours the growth is secondary, and an index of generalisation of disease, starting in some organ more or less remote from the liver. In most cases of primary cancer of the liver there is a local multiplicity—the growths being disseminated through the parenchyma of the gland. It is a necessary condition of success for the removal of any hepatic tumour that the growth be a single one, and also that it be situated at a part of the liver that is readily accessible. It would not be justifiable to attack any tumour deeply situated in either of the two large lobes of the liver. A condition favouring extirpation would be the presence of a pedicle. The removal of a tumour presenting these favourable conditions for operative treatment would still be contra-indicated if such tumour has contracted close adhesions with the abdominal wall or the surrounding abdominal viscera. The prospects of success are least favourable in cancer cases on account of the probability of a recurrence. In the liver, however, as in other organs, a centre of infection constituted by a malignant growth ought to be suppressed, and even in cases in which extirpation would be impossible a palliative operation—cholecystostomy, for example—would be indicated, with the object of relieving functional disturbances, and so of prolonging the life of the patient. Keen[2] relates his conclusions as regards the removal of an angeioma of the liver by elastic constriction external to the abdominal cavity as follows: (1) Tumours of the liver, and even large portions of the liver, can be removed without undue disturbance of its functions. (2) That the escape of bile into the peritoneal cavity is not usual after such an operation, and even if it occurs fresh bile is not infective, and therefore does not produce peritonitis. (3) The removal of a tumour can be done by ligation, by blunt dissection, by the cautery, by the knife or scissors, or by a combination of these methods. If the base is very large or the tumour very vascular, an artificial pedicle can be made by the cautery and an elastic ligature applied. (4) In case a syphilitic tumour is suspected, no operation should be done until after full trial of anti-syphilitic treatment has been made and failed.

**Abscess of the Liver** should be treated, according to Fontan,[3] by the following method: (1) A very large opening should be made in the region where the presence of pus has been made out. (2) The costal pleura should be sutured to the diaphragmatic pleura in cases in which the chest is opened, and the parietal peritoneum to the hepatic peritoneum when the abdominal cavity alone is involved. (3) Methodical and prudent, though complete, curettage of the wall of the abscess cavity should be done. The author endeavours, when possible, to perform a transpleural operation, and considers that the seat of election should be the axillary line, or a little behind this at the level of the eighth, ninth, or tenth rib, of which a piece from six to eight centimetres in length should be resected. Curettage removes from the cavity the detritus and small masses of dead tissue, and is not likely to cause either hæmorrhage or a free discharge of bile, as the blood vessels and biliary ducts of the walls of the sac are closed by thrombosis. The danger of aspirating the liver in such cases for purposes of diagnosis is dwelt on by Hatch,[4] who says it should never be used for such a purpose, and quotes several fatal cases resulting therefrom.

**Cholelithiasis.**—The indications for the various methods of operating are based upon the following principles by Forgue[5]: (1) Cholecystostomy should be performed where the biliary passages, either direct or indirect, obstructed by calculi or not, are the seat of inflammation which produces intense febrile movements, continuous or with exacerbations. (2) Cholecystectomy is the operation of choice in cases of biliary calculi. There must, however, be asepsis of the entire tract, and the permeability present or attainable of the ductus choledochus. (3) Cholecystenterostomy is most favourably employed in cases where an exploratory laparotomy shows an inoperable neoplasm compressing the ductus choledochus. The operation is favoured in these cases by the dilatation of the duct. There are certain other cases where the gall-bladder is contracted and the duct obstructed by a firmly implanted calculus. (4) Choledochotomy is the operation preferable in cases of obstruction of the ductus choledochus by a calculus, but in these cases a preliminary cholecystostomy is generally a necessity. Donald[6] reports a case of cholecystostomy for gall stones in which an hour-glass contraction of the gall-bladder was present; and other cases of choledochotomy, cholecystostomy, &c., are reported by Briddon[7] and Brown.[8]

**Rupture of Gall-Bladder.**—A most interesting case of this condition is reported by Martin,[9] who performed laparotomy 24 days after the injury, which was caused in a boy of nine years by a cart, weighing 15 cwt., passing over his abdomen. At the time of operation nearly five pints of bile-stained fluid escaped from the peritoneal cavity. The boy made a good recovery.

**Splenectomy for Ruptured Spleen** is considered by Ballance,[10] who quotes several cases without external wound. The diagnosis of this condition is arrived at from (1) The locality of the injury; (2) the evidence of internal hæmorrhage; (3) the large *fixed* dulness in the left flank. The fixed dulness in the left loin and splenic region is not present in intra-abdominal hæmorrhage from other organs, and is caused by this region being occupied by large quantities of clot. The dulness, therefore, cannot change with position, and is pathognomonic of this injury. The unusually large proportion of white corpuscles in splenic venous blood offers an explanation of the local coagulation of the effused blood in splenic rupture, and of the fact that these patients do not readily bleed to death. In slight splenic ruptures bleeding is probably quickly and permanently arrested by nature's method of coagulation; but when the injury is severe, and bleeding at first ceases, it will probab y recommence as soon as the patient has rallied. A patient with a belly full of blood is in the gravest danger, and no method (suture of rent, packing with gauze, &c.) less radical than excision can unequivocally be relied upon to arrest the hæmorrhage. The rhythmic contractions and expansions of the spleen add to the difficulties and dangers of the arrestment of hæmorrhage by any other plan than that of excision. The symptoms produced by the excision of the normal spleen in the adult man are: (1) Progressive loss of strength and weight. (2) Extreme anæmia. (3) A daily rise of temperature. (4) Increased frequency of pulse. (5) Fainting attacks, with great pallor of surface. (6) Headache, drowsiness, thirst, voracity. (7) Diminished or increased secretion of urine. (8) Griping pains in abdomen and tenderness along the long bones. (9) Enlargement of lymphatic glands. (10) Certain typical blood changes. These symptoms become manifest some ten to fourteen days after operation, continue in great severity for a fortnight, and then a slow but perfect convalescence ensues. These symptoms are relieved by various remedies—fresh sheep's spleens lightly grilled, or extract of fresh spleen made with normal saline solution; red bone marrow mixed with anchovy paste to make it palatable; arsenic; and cod liver oil. Other cases of splenectomy are recorded by V. Beck[11] and by McBurney.[12] Tedeschi[13] discusses the question as to whether new splenic tissue is formed after removal of the spleen, and is inclined to think it is. He quotes the case of a girl in which the post-mortem examination showed no spleen in its normal situation, but found numerous nodular tumours of various size which were composed of splenic tissue. The author explains these aberrant splenic structures by a dislocation of the true spleen, with torsion of its pedicle; in this wise most of the splenic structure would be destroyed, and its function would be replaced by the growth of these spleens arising from the peritoneum. Similar masses of splenic tissue are found in animals after experimental splenectomy, and he concludes that these are not supernumerary spleens, but really spleens newly grown after the operation, because supernumerary spleens are generally only two or three in number; the latter organs, too, are found in the gastro-splenic ligament at the side of the true, normally located spleen; while those that grow after operation are found either in the great omentum or in duplications of the peritoneal folds.

[1] Brit. Med. Jour., May 28, 1898; and Rev. de Chir., May, 1898. [2] Therap. Gaz., Jan., 1898, p. 53. [3] Brit. Med. Jour., April 23, 1898; and Bull. et Mém. de la Soc. de Chir., No. 7, 1898. [4] Lancet, May 14, 1898; and Indian Med. Gaz., April, 1898. [5] Amer. Jour. Med. Sci., June. 1898, p. 729. [6] Glasgow Med Jour., May, 1898, p. 348; and Brit. Med Jour., May 7, 1898, p. 1196. [7] Annals of Surg., April, 1898, p. 529. [8] Ibid., p. 535. [9] Lancet, May 21, 1898. [10] Practitioner, April, 1898, p. 347. [11] Med. Rec., May 21, 1898. [12] Ibid., April 23, 1898, p. 601. [13] Ibid., April 23, 1898, p. 591.

### SURGERY OF THE BRAIN.

**Injuries.**—In reporting several interesting cases of brain injury, Van Santvoord[1] gives a brief *résumé* of the current views on the "mechanism of the production of cerebral lesions by blunt violence." His paper, so far as this subject is concerned, is simply a repetition of Duret's well-known theory of the disturbance of the cerebro-spinal fluid being the determining factor in the production of brain lesions, to which, apparently, the writer subscribes.

Referring to some points in the pathology and symptomatology of brain injuries, the writer says: (1) That primary unconsciousness after an injury to the brain may be entirely due to shock, *i.e.*, reflex contraction of the cerebral arteries. This is rarely fatal, and, if uncomplicated, is usually recovered from in a few minutes or hours. Apparent complete recovery, however, does not exclude the possibility of the existence of latent lesions, which may manifest their existence later. (2) Sudden or rapid insensibility after recovery of consciousness indicates hæmorrhage. (3) Prolonged unconsciousness without elevation of temperature means compression from hæmorrhage, either epidural or subdural. (4) The absence of fever does not necessarily exclude laceration. (5) Prolonged unconsciousness with elevation of temperature indicates grave lesion of the brain substance, with or without considerable hæmorrhage. (6) The suppression of the function of a limited portion of the brain by laceration, unless involving some vital spot, such as the medulla, is not a serious matter, as regards life, at least. Such lacerated portions may, however, when undergoing secondary softening, give rise to grave or fatal secondary hæmorrhages, or may be the point of departure of secondary inflammation. In these latter cases the multiple minor lesions scattered widely through the brain, comprehended under the term diffuse cerebral contusion, are often pathologically of more importance than the few or isolated foci of extensive laceration. (7) The elevation of temperature associated with these lesions has been attributed to injury to the thermic centres, but this theory fails to explain the post mortem rise which so often takes place. (8) The diffuse cerebral hyperæmia, with or without œdema found after death, is not associated with cellular infiltration. It is probably inflammatory, and related to the acute serous meningitis which follows bacterial infection. Alcoholism may be a factor in its production. (9) Death in head injuries is due to gross lesions of vital centres, compression from primary or secondary hæmorrhages, the secondary effects of diffuse cerebral contusion, or inflammation from infection. (10) In compression from hæmorrhage surgery may be of use; and in cases of serous effusion lumbar tapping promises some benefit.

Mr. Herbert W. Page[2] makes two exceedingly interesting cases of head injury the text of some instructive remarks on this subject. The first case was that of a railway guard, aged 49, who was struck on the forehead by the open door of a railway carriage in motion. He was knocked backward and struck the back of his head on the platform. He was collapsed and dazed, but not unconscious, when admitted to hospital. Gave his name and address. Respirations 20 and pulse 90. The only sign of cranial fracture was the escape of blood and cerebro-spinal fluid from the left ear. The pupils were equal and reacted to light sluggishly. An interesting point in the case is that within three-quarters of an hour of his admission he had become so much worse that he was profoundly comatose; clonic movements of the left arm gave place very soon to spastic rigidity, and this rapidly involved all the limbs. The pulse and respiration were quickly failing, and the pupils became fixed—the left in dilatation. The skull was rapidly opened over the left middle meningeal artery; but it was found that the blood was inside the dura and most abundant at the base. A considerable quantity of blood was evacuated by means of a tube and aspirator, with very marked benefit to the symptoms. Pulse and respiration improved, and the left pupil again corresponded in size to the right. It was obvious that serious damage had been done to the base of the skull, and that bleeding was taking place from an inaccessible vessel in that region. The man died a few hours later. Post-mortem examination confirmed the diagnosis. The sub-arachnoid space all over the brain contained blood, but this was especially abundant at the base. The base of the brain was extensively lacerated, and much damage was done to the skull.

The second case was that of a man aged 50 who was struck on the back by the buffer of a train, his head striking some iron girders when he fell. On admission he was half unconscious, and brain matter was protruding through a severe comminuted and depressed fracture of the left side of the vault of the skull. He was hemiplegic on the right side; his breathing was entirely diaphragmatic, his pupils unequal, the left the larger, both reacted to light. Operation was performed, but he died three days later without having regained consciousness. On post-mortem examination it was found that the skull and cortex had borne the brunt of the damage, the internal parts of the brain being fairly normal.

Referring to these two cases, the writer draws attention to the following facts of clinical importance: (1) With aseptic precautions it is always advisable to operate for the removal of useless or displaced portions of bone. (2) In basal fractures direct external evidence of damage to bone is seldom forthcoming, and operative interference is not often of benefit. (3) Rapid (or gradual) increase of unconsciousness is a clear indication for operation, and in a considerable proportion of cases the bleeding takes place from the middle meningeal artery, dilatation of the pupil on the affected side being corroborative evidence. (4) Increased experience shows that classical signs in cranial injuries are often belied. Each case must be studied on its own merits, and in many only an approximate diagnosis is possible. (5) On the whole it is better to operate in severe head injuries when in doubt than to withhold from doing so.

Scudder[3] reports a case of operation for middle meningeal hæmorrhage following an accident in a woman of 69, which resulted in recovery.

[1] Med. Rec., April 30, 1898. [2] Lancet, April 30, 1898. [3] Med. Rec.' June 18, 1898.

